# Supplementary material for: Cornuside Is a Potential Agent against Alzheimer’s Disease via Orchestration of Reactive Astrocytes
Source: Nutrients. 2022 Aug 3;14(15):3179. doi: 10.3390/nu14153179 (PMC9370780; doi:10.3390/nu14153179)
Supplement: Supplementary file 1 [file nutrients-14-03179-s001.zip › nutrients-1797854-supplementary.pdf]

Supplementary materials

Cornuside is a Potential Agent against Alzheimer's Disease via Orchestration of Reactive Astrocytes

1 q-PCR primer sequences

Supplementary Table S1 q-PCR primer sequences

Gene	Forward (5'-3')	Reverse (5'-3')
AQP4 (mouse)	CTCCCTTTGCTTTGGACTCA	CGATGCTGATCTTTCGTGTG
GFAP (mouse)	GCCACCAGTAACATGCAAGA	GGCGATAGTCGTTAGCTTCG
VIM (mouse)	CTGCACGATGAAGAGATCCA	AGCCACGCTTTCATACTGCT
S100B (mouse)	GGTTGCCCTCATTGATGTCTTC	TCCTGCTCCTTGATTTCCTCC
Psmb8 (mouse)	TCATCGTGGCGGTGGACTCC	CTGACAGTCGGCTGCACAACC
Serping1 (mouse)	GCTCTACCACGCCTTCTCAG	GGATGCTCTCCAAGTTGCTC
Complement C3 (mouse)	CCAGCTCCCCATTAGCTCTG	GCACTTGCCTCTTTAGGAAGTC
Amigo2 (mouse)	GAGGCGACCATAATGTCGTT	GCATCCAACAGTCCGATTCT
Gbp2 (mouse)	GGAGGAGCTGTGTGGTGAAT	TTAGACGTGGCCCATTGACT
Ptx3 (mouse)	TTCCTGAGGGTGGACTCCTA	CCGATCCCAGATATTGAAGC
S100A10 (mouse)	GTGCTCATGGAACGGGAGT	AAAGCTCTGGAAGCCCACTT
Tgm1 (mouse)	CCCTGGATGACAATGGAGTT	GAATAGCCGGTGCGTAGGTA
Ptgs2 (mouse)	CGGACTGGATTCTACGGTGA	CCCTTGAAGTGGGTCAGGAT
CD14 (mouse)	CCCAAGCACACTCACTCAAC	ATCAGTCCTTTCTCGCCCAA
BDNF (mouse)	GCGGCAGATCCCCCGACTGC	AAGTTGTGCGCAAATGACTG

TGF-β (mouse)	CTCCCGTGGCTTCTAGTGC	GCCTTAGTTTGGACAGGATCTG
Thbs1 (mouse)	TGATGACTACGCTGGCTTTG	TGAGTATCCCTGAGCCCTTG
GAPDH (mouse)	TCACCATCTTCCAGGAGCGAG AC	AGACACCAGTAGACTCCACGA CATAC
Complement C3 (rat)	TTGTCCCCTTGAAGATCGGC	TCATTCCTTCTGGCACGACC
S100A10 (rat)	CACACCTTGATGCCGTCCTCT	GGCAACCGGATTGCAAACAAT
GAPDH (rat)	ACAAGCAACAGGGTGGTGGAC	TTTGAGGGTGCAGCGAACTT

2 Chemical structure of Cornuside

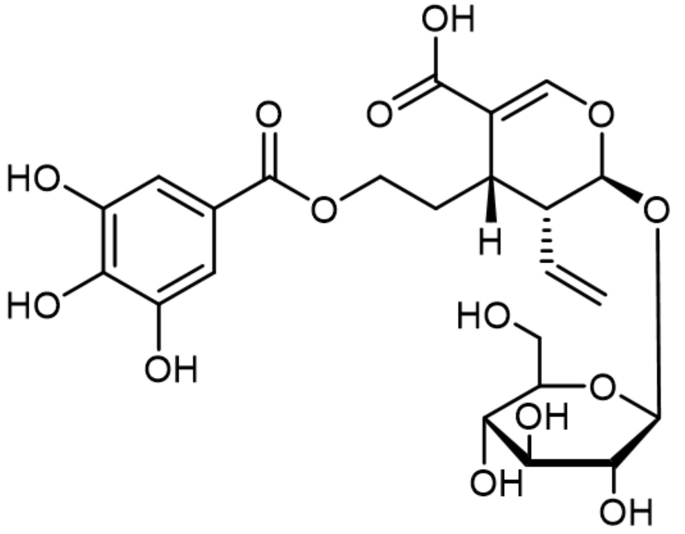

Supplementary Figure S1 Chemical structure of Cornuside

3 Preparation of MCM

BV2 cells were cultured in the DMEM serum-free medium containing LPS (0, 0.1, 0.5, 1, 2, 4 μg/mL) and incubated for 24 h. The microglia conditioned medium (MCM) was then collected. We then determined the LPS concentration for MCM based on the NO content detected by Nitric Oxide (NO) assay kit (Nitrate reductase method). The result showed that the content of NO peaked in MCM with LPS treatment at the

concentration of 2 μg/mL, which was further used for preparation of MCM. Next, we introduced LPS (2 μg/mL) to treat microglia in the DMEM serum-free medium. After incubation for 24 h, the cell supernatant was collected and centrifugated. ELISA was performed to detect the concentration of IL-1β, TNF-α and IL-6 in the cell supernatant. The results showed that the concentrations of IL-1β, TNF-α and IL-6 were significantly higher in MCM than those in control media.

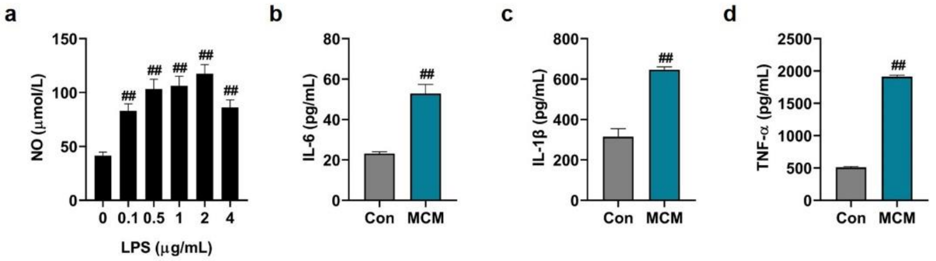

Supplementary Figure S2 The levels of pro-inflammatory cytokines in MCM

a The NO content in MCM with or without LPS treatment as indicated. **b** The levels of IL-16 **c** IL-1β and **d** TNF-α in MCM. All values are presented as mean ± SEM, n = 3 per group. ## $p < 0.01$ versus Con.

4 The effect of cornuside on cell viability in C6 cells

MTT assay was used to detect the effects of cornuside at different concentrations on the viability of C6 cells. There was no significant difference in cell viability at dosage below 40 μmol/L cornuside. Cornuside (10 μmol/L) was used for the following experiments.

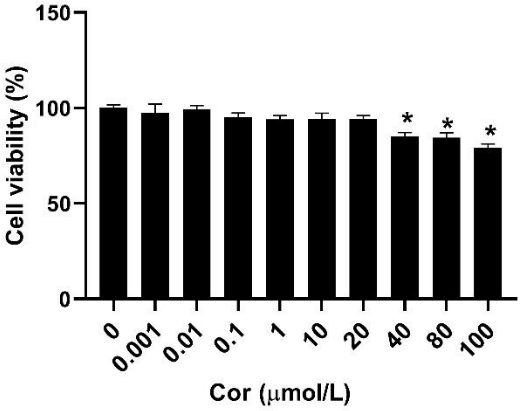

Supplementary Figure S3 The effect of cornuside on cell viability in C6 cells

All values are presented as mean ± SEM, n = 3 per group. * $p < 0.05$ versus Con.

5 The interference efficiency of Nrf2 siRNA

Three pre-designed siRNA directed against Nrf2 were used to down-regulate Nrf2, and a negative control siRNA (NC) was used to monitor the off-target effect. 72 h after the transfection, the knockdown efficiency of Nrf2 at the protein level was evaluated by western blot. The results showed that the 3$^{\#}$ Nrf2 siRNA interference efficiency was the most distinctive, which was used for the following experiments.

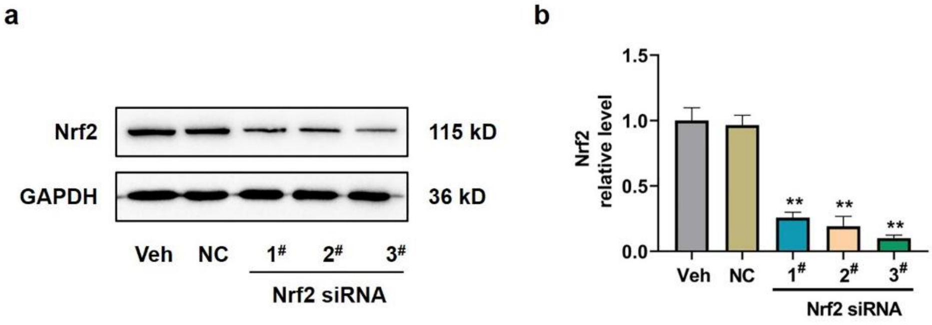

Supplementary Figure S4 The interference efficiency of Nrf2 siRNA

a The representative blotting images and **b** Quantification analysis of Nrf2 protein. All values are presented as mean ± SEM, n = 3 per group. ** $p < 0.01$ compared with NC group.
